# Organic resources from Madagascar: Dataset of chemical and near-infrared spectroscopy measurements

**DOI:** 10.1016/j.dib.2022.108350

**Published:** 2022-06-02

**Authors:** Nantenaina Rabetokotany Rarivoson, Tantely Razafimbelo, Dominique Masse, Heriniaina Ramahefarison, Laurent Thuriès

**Affiliations:** a Ecole Supérieure Polytechnique d'Antananarivo, Université d'Antananarivo, BP 1500, Madagascar; b Laboratoire des Radioisotopes, Université d'Antananarivo, Madagascar; c IRD, Eco&Sols, Abidjan, Côte d'Ivoire; d Université de Mahajanga, Madagascar; e CIRAD, Recyclage et Risque, Saint-Denis, Réunion, France

**Keywords:** Agro-industrial waste, Livestock manure, Urban waste, MSW compost, VIS-NIR spectroscopy, Madagascar

## Abstract

Organic wastes originating from livestock, agro-industry or urban activities may represent true resources when recycled for new uses, for example, as soil improvers, organic fertilizers or bioenergy sources. The compositional characteristics of these organic resources (ORs) can vary considerably depending on origin, nature, processing, stage, and state. Despite being of potential interest to different stakeholders in a circular economy, the variability in OR characteristics and the difficulty of accessing reliable, fast and inexpensive analysis methods may curb the recycling of OR in the agriculture or bioenergy sectors. As is the case in other low-income countries, scarcity of data on OR characteristics and the difficulty in assessing these data (due to cost and the sparsity of laboratories) is particularly acute in Madagascar, thus impairing the rational utilization of OR in the agricultural or bioenergy sectors. Visible-near infrared spectroscopy (VIS-NIR) has proven to be suitable for the fast, reliable and low-cost determination of the composition of different ORs, usually through the development of calibration models based on one type of OR by single research or lab groups. It is challenging to develop VIS-NIR models based on several types of ORs encompassing a wide range of target characteristics. Another challenging issue is the extension of databases containing spectra acquired on different spectrometers to increase model genericity. In both cases, standardization can be performed to resolve the problem of developing models for diverse ORs whose spectra originate from different laboratories. To assess the ability to develop VIS-NIR models with as much genericity as possible, we built a large database containing a wide diversity of ORs produced in Madagascar. The data presented in this paper were obtained by chemical and spectral analyses of 1,000 ORs collected from five districts in Madagascar. The data are accompanied by fine-grained metadata defined by 32 descriptors of ORs, including origin (animal, agro-industrial, and urban); nature (manure, agro-industrial waste, and compost); farm type (smallholder and agricultural factory); exploitation type (smallholder farm, factory farm, on-farm compost facility, and town compost facility); diversity of animal feed, litter, sex, and age; and diversity of bedding material. The chemical properties (including the organic nitrogen, organic carbon, organic matter, inorganic matter, phosphorus, potassium, calcium, magnesium, zinc, copper, nickel, chromium, cadmium, and lead and soluble, hemicellulose, cellulose, lignin and cutin fractions) were analyzed following laboratory standards. The number of analyses performed ranged from 39 to 180 depending on the chemical property. VIS-NIR spectra were acquired using a Labspec spectrometer. To facilitate the merging of spectral data or the development of VIS-NIR models based on broad datasets, the spectra were presented in raw form and after standardization. The dataset is original in terms of sources and width. This dataset should be of particular interest to chemometricians, biogeochemists, agronomists, energy planners, hygienists and other professionals involved in recycling ORs for various new purposes in low-income countries and elsewhere.

## Specifications Table

**Table:** 

Subject	Analytical Chemistry; Analytical Chemistry: Spectroscopy
Specific subject area	Compositional and spectroscopic analysis of organic resources with animal, agro-industrial, and urban origins collected from different regions of Madagascar
Type of data	Table Graphs Spectroscopic data
How data were acquired	Absorbance spectra of samples were acquired by a LabSpec VIS-NIR spectrometer (ASD, Boulder, USA).Conventional laboratory analyses (according to standardized methods from the French standards bureau, AFNOR) were performed to determine the total organic matter (TOM), total organic carbon (TOC), inorganic matter (Ash), total nitrogen (TN), phosphorus (totP), calcium (totCa), magnesium (totMg), potassium (totK), biochemical fractions (soluble, hemicellulose, cellulose, lignin and cutin), and contents of cadmium (Ca), chromium (Cr), copper (Cu), lead (Pb), nickel (Ni), and zinc (Zn).
Data format	Raw Data
Description of data collection	The collection procedure was adapted according to the nature and size of the sites (ranging from small barns to large municipal solid waste landfills). Sample collection consisted of the selection of representative zones, adaptation of the number of subsamples to be collected (a minimum of 3 to 20 subsamples) and the creation of composite samples from the subsamples.Samples were rapidly transported to the laboratory and prepared for spectra acquisition (dried ground state) and compositional analysis (fresh or dried ground states according to the type of analysis).The 1,000 dried ground samples were scanned with a portable LabSpec spectrometer (ASD, Boulder, USA) to acquire VIS-NIR spectra. Chemometric tools were used to standardize the spectral data, using an XDS spectrometer (Foss, Silver Spring, USA) as the reference instrument.
Data source location	Laboratoire des Radioisotopes, BP 3383, Route d'Andraisoro, 101 Antananarivo, Madagascar
Data accessibility	Repository name: https://dataverse.cirad.fr/Data identification number (permanent identifier, i.e. DOI number):10.18167/DVN1/YRMQ52and10.18167/DVN1/SZSTBDDirect link to the dataset:https://dataverse.cirad.fr/dataset.xhtml?persistentId=doi:10.18167/DVN1/YRMQ52andhttps://dataverse.cirad.fr/dataset.xhtml?persistentId=doi:10.18167/DVN1/SZSTBD

## Value of the Data

• Wide-scaled metadata (e.g., the OR origin) to fine-scaled metadata (e.g., the nature of the animal feed) provide valuable information given the scarcity of such data in low-income countries, including Madagascar. This dataset consists of visible-near infrared spectra and the chemical compositions of a large collection (1,000 samples) of organic resources of animal, agro-industrial, and urban origin collected from six regions of Madagascar. The data on organic resources include the origin (animal, agro-industrial, and urban); nature (manure, agro-industrial waste, and compost); farm type (smallholder and agricultural factory); exploitation type (smallholder farm, factory farm, on-farm composting facility, and town composting facility); diversity of animal feed, litter, sex, and age; and diversity of bedding material.
• Unstandardized or standardized spectral data combined with compositional data are useful to chemometricians and theme specialists (e.g., the fertilization value for agronomists, digestibility for zootechnicians, energy potential for energy planners, and the characteristics of untreated vs. composted waste for hygienists) for calibrating NIR-based models used in rapid compositional analysis.
• VIS-NIR spectra of standard cells are provided in raw and standardized forms to enable the extension or merging with existing databases.

## 1 Data Description

In this article, the chemical composition and NIR spectral data of 1000 OR samples collected from different regions of Madagascar are presented as a table and 6 figures. The dataset covers a broad range of sampled raw or transformed organic materials. These materials include 863 animal manures (piglet, pig, barrow, sow, broiler, chick, hen, cow, heifer, zebu bull, rabbit, pigeon, and sheep); 81 urban wastes (raw municipal waste, composted municipal waste, fine fraction from municipal waste compost stockpiled from 6 months to 10 years, rice husk ashes, and vermicompost); 36 agro-industrial byproducts (bone, horn, blood, shell, cotton cake, peanut cake, groundnut cake, and tobacco ash) and 20 mixed wastes (mixtures of animal, urban and/or agro-industrial origins).

Table 1 summarizes the contents of organic nitrogen, organic carbon, organic matter, inorganic matter (ash), phosphorus, potassium, calcium, magnesium, zinc, copper, nickel, chromium, cadmium, lead, and Van Soest [1] fractions (soluble, hemicellulose, cellulose, and lignin) for the samples. The number of analyzed samples depended on the chemical component and ranged from 39 for trace metals to 180 for organic carbon or organic nitrogen.

**Table 1:** Statistics for chemical properties of organic residues (expressed on a dry matter basis).

Properties		Unit	n	Mean	SD	Min	Max	Range
Total nitrogen	TN	g.kg^−1^	180	21.98	13.97	0.0001	99.20	99.20
Total organic carbon	TOC	g.100 g^−1^	180	23.72	9.87	2.56	47.25	44.69
Total inorganic matter	Ash	g.100 g^−1^	87	55.81	22.10	2.75	98.50	95.75
Total phosphorus	totP	g.kg^−1^	88	13.23	19.76	0.24	158.2	157.9
Total potassium	totK	g.kg^−1^	88	14.28	16.58	0.65	151.6	150.9
Total calcium	totCa	g.kg^−1^	66	42.98	39.49	0.20	137.7	137.5
Total magnesium	totMg	g.kg^−1^	67	7.66	4.35	0.68	23.12	22.43
Zinc	Zn	mg.kg^−1^	39	559	576	39.38	2220	2181
Copper	Cu	mg.kg^−1^	39	109	150	5.51	540	535
Nickel	Ni	mg.kg^−1^	39	23.29	18.13	3.56	64.13	60.57
Chromium	Cr	mg.kg^−1^	39	84.18	80.38	4.83	293	288
Cadmium	Cd	mg.kg^−1^	39	0.97	0.81	0.0001	3.28	3.28
Lead	Pb	mg.kg^−1^	39	429	806	0.0001	3982	3982
Soluble	SOL	g.100 g^−1^	87	17.19	10.80	0.14	71.24	71.10
Hemicellulose	HEM	g.100 g^−1^	87	9.34	7.22	0.0001	31.17	31.17
Cellulose	CEL	g.100 g^−1^	87	9.86	8.34	0.0001	45.38	45.38
Lignin & cutin	LIC	g.100 g^−1^	87	7.83	7.26	0.08	39.43	39.35

Table 1 presents the mean, standard deviation, minimum, maximum and range for the aforementioned properties.

**Fig. 1:**
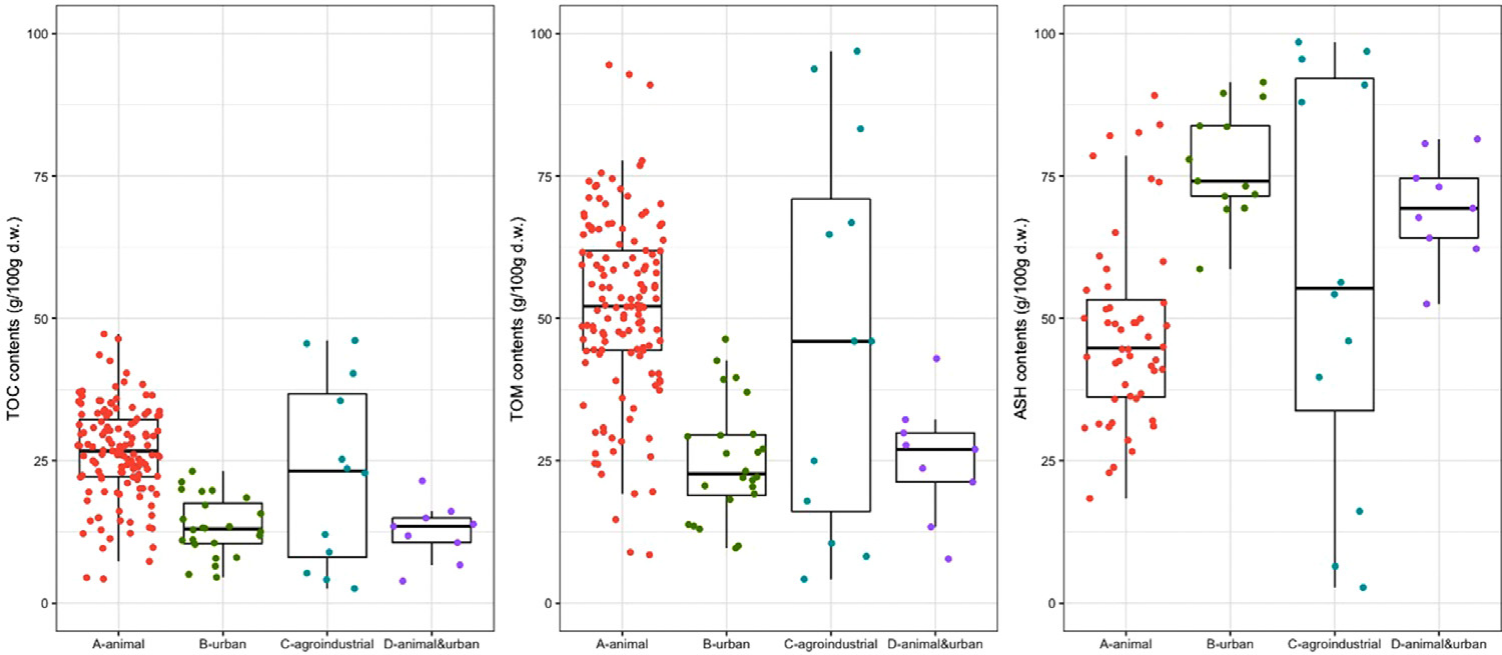
Contents of total organic carbon (TOC), total organic matter (TOM), and total inorganic matter (ASH).

**Fig. 2:**
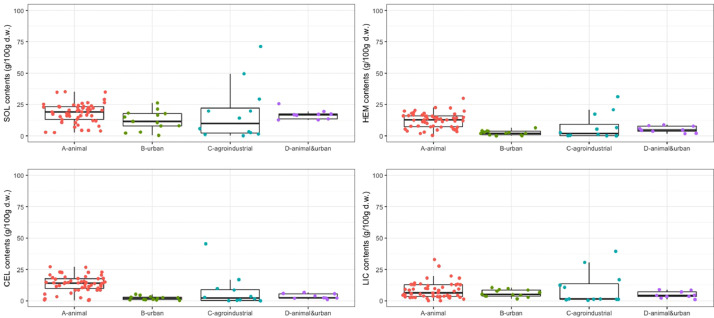
Contents of biochemical fractions (soluble, hemicellulose, cellulose, lignin and cutin: SOL, HEM, CEL, and LIC, respectively).

**Fig. 3:**
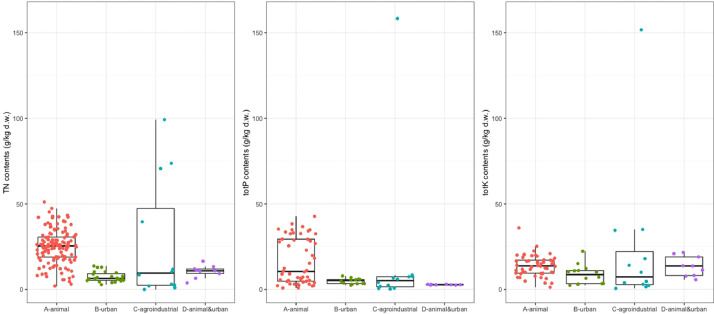
Contents of total nitrogen (TN), total phosphorus (totP), and total potassium (totK).

**Fig. 4:**
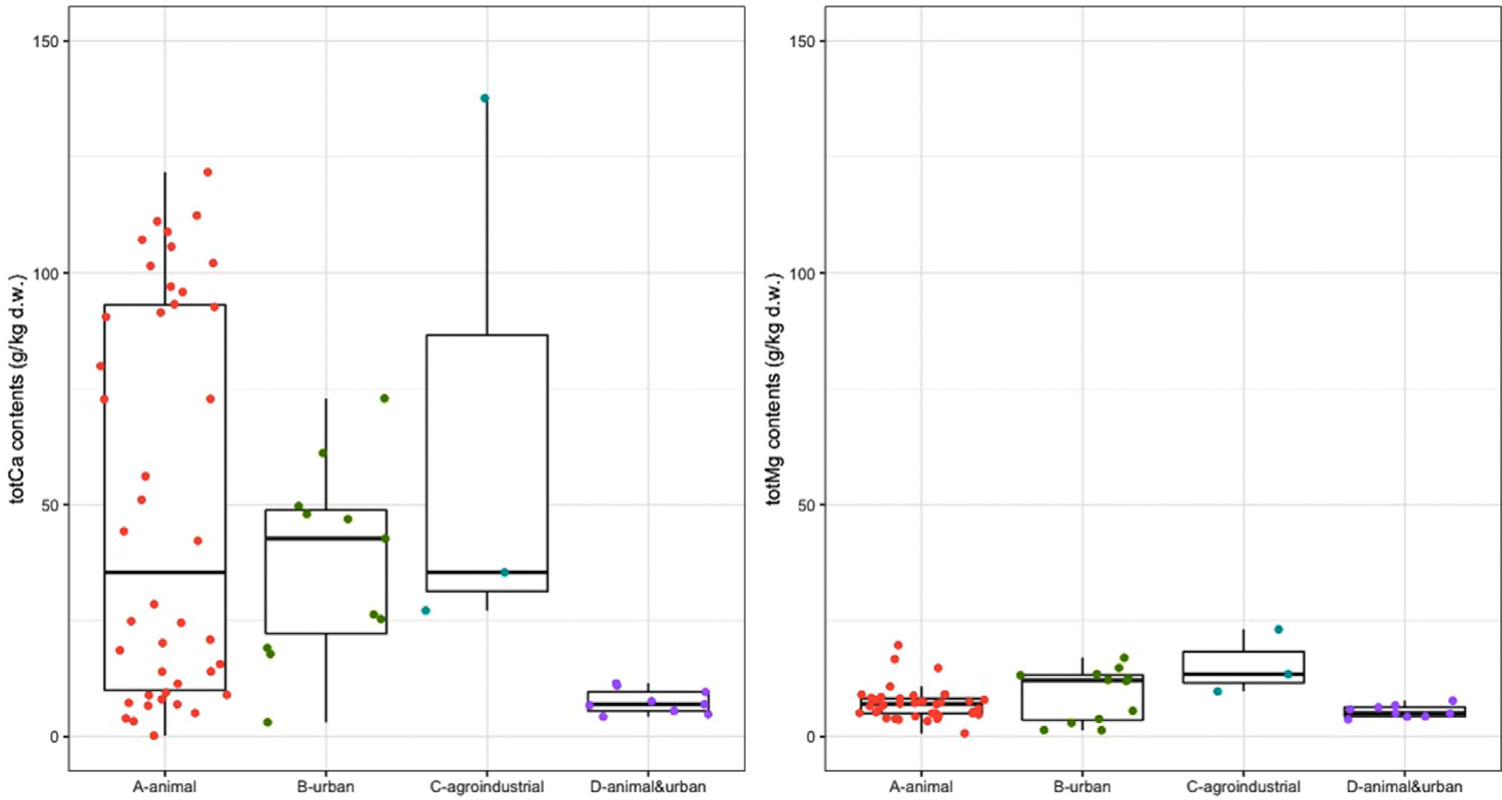
Contents of total calcium (totCa), and total magnesium (totMg).

**Fig. 5:**
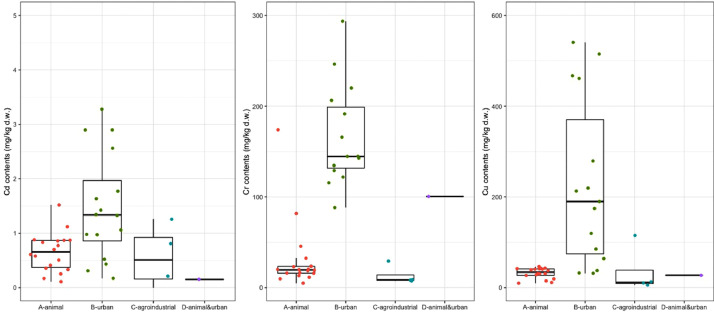
Contents of trace elements: Cadmium (Cd), Chromium (Cr) and Copper (Cu).

**Fig. 6:**
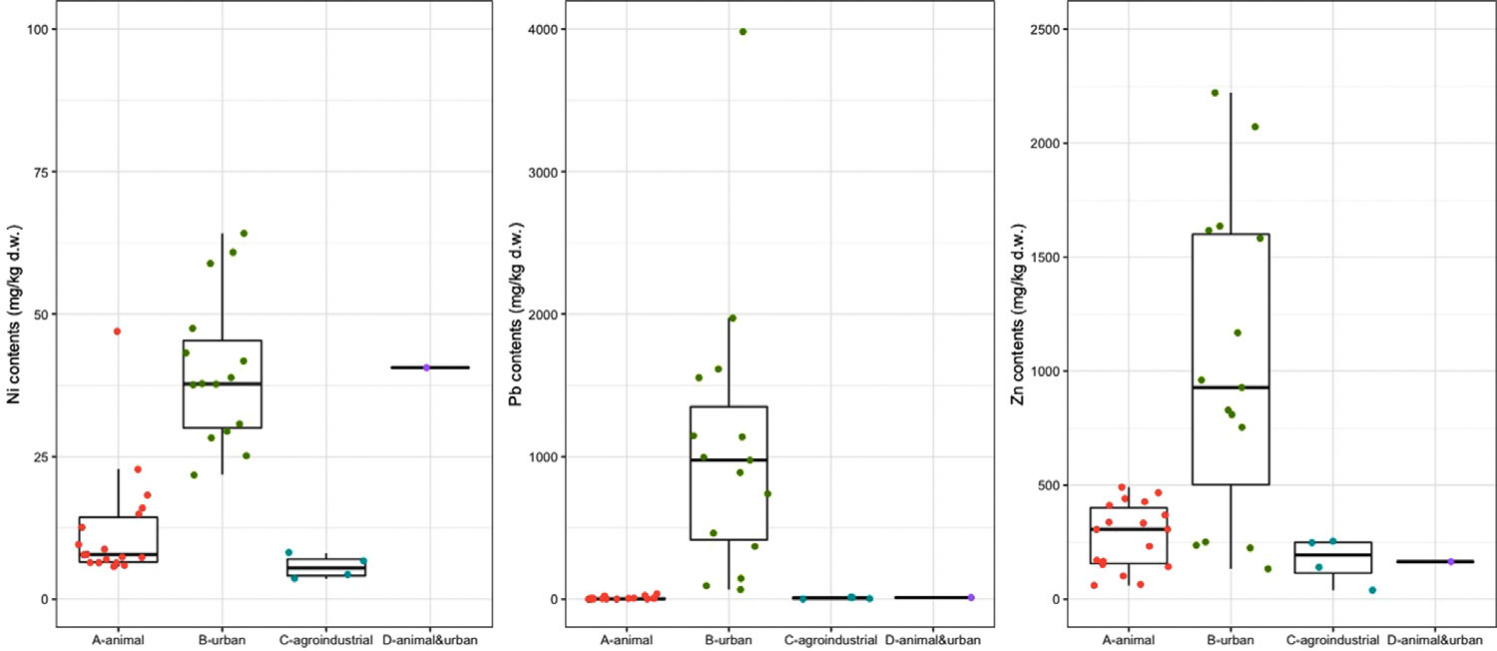
Contents of trace elements: Nickel (Ni), Lead (Pb) and Zinc (Zn).

Fig. 1, Fig. 2, Fig. 3, Fig. 4, Fig. 5, Fig. 6 are boxplots of the data obtained from compositional analyses of ORs with different origins. These figures highlight the variability in the OR composition.

**Fig. 7:**
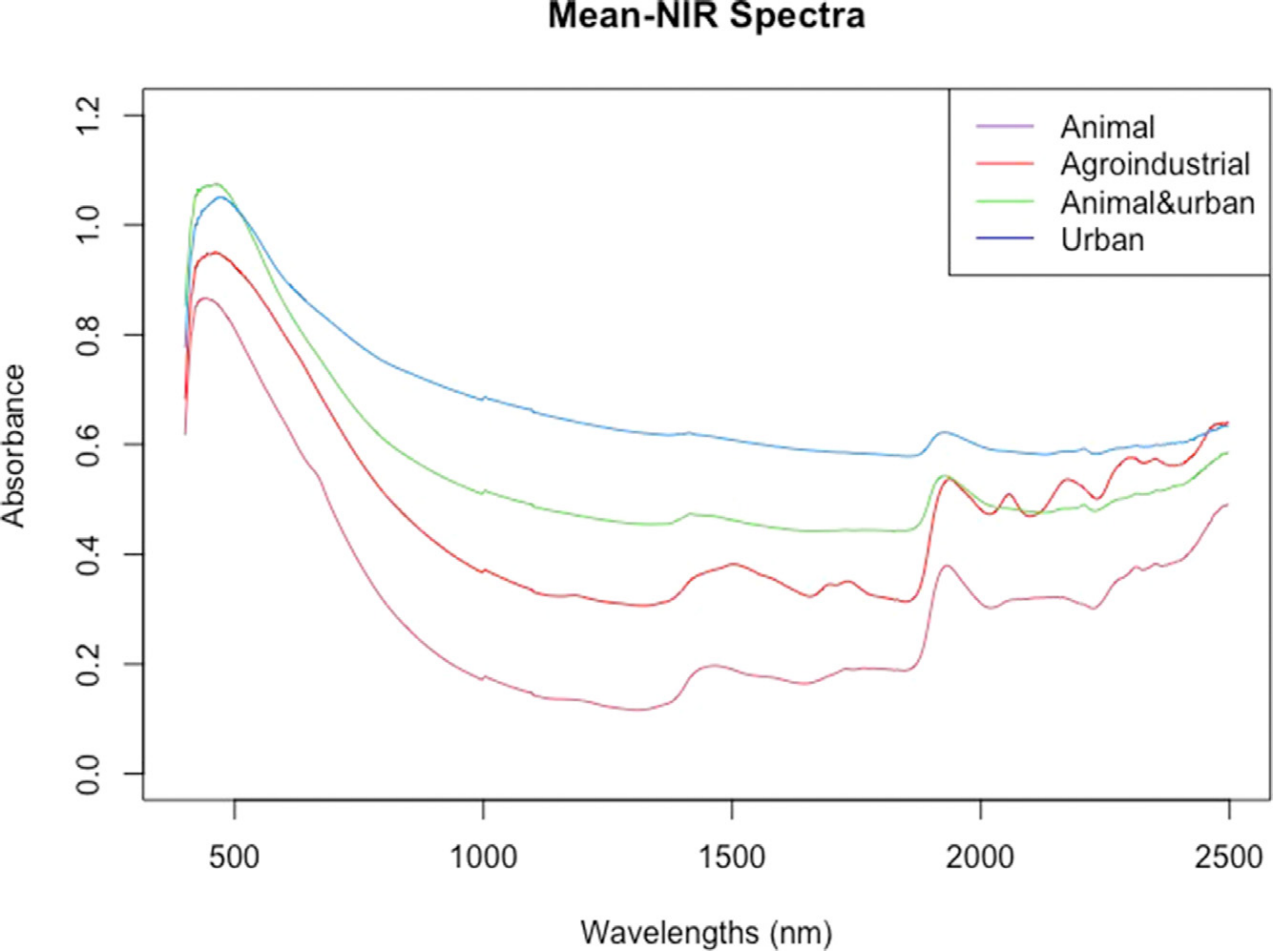
Mean VIS-NIR standardized spectra for various categories of organic resources.

**Fig. 8:**
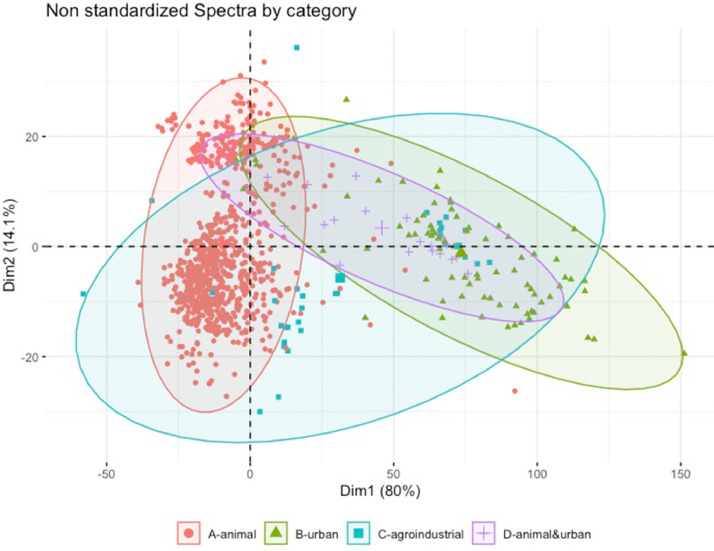
Projection of the first two principal components of a PCA of 1,000 VIS-NIR spectra.

Fig. 7 displays the mean VIS-NIR spectra by nature (manure, agro-industrial, urban, and manure+urban mixtures), and Fig. 8 presents the projection of the two first principal components of a PCA of the VIS-NIR spectra.

**Fig. 9:**
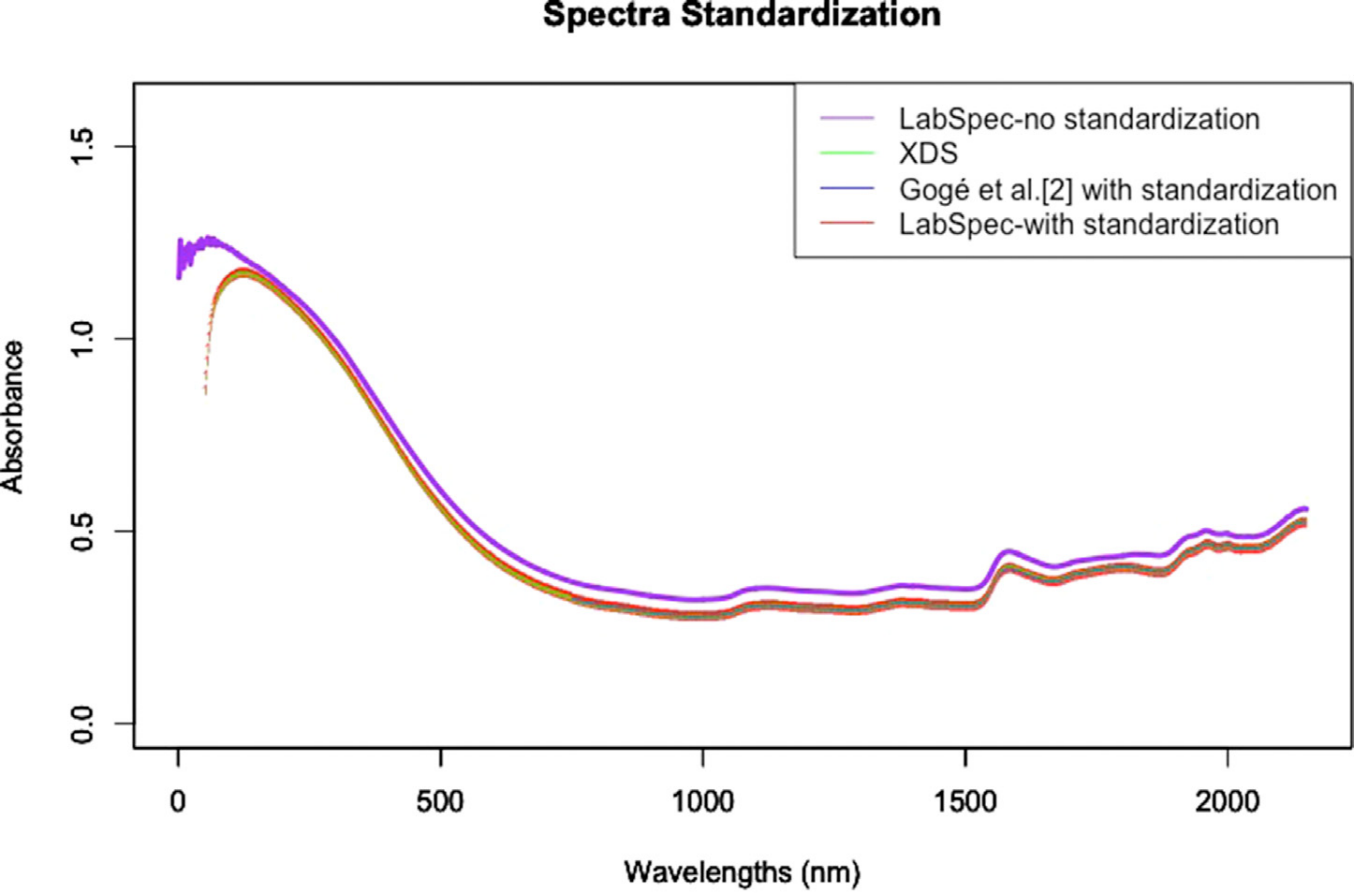
VIS-NIR spectra of a standard cell (green waste compost named “2b” in Gogé et al. [2]). Reported spectra are obtained using a Labspec spectrometer, an XDS reference spectrometer, a Labspec spectrometer after standardization based on using an XDS spectrometer as the reference NIR spectrometer, and the same XDS spectrometer by Gogé et al. [2].

Fig. 9 displays the VIS-NIR spectra of the standard cells used to standardize the NIR spectra.

The dataset is composed of 6 MS Excel® files of VIS-NIR data (OR and standard cells) and 2 MS Excel® files for the metadata and compositional analyses. The metadata file contains 32 descriptors, including the sample identification number; VIS-NIR spectrometer type; sampling location (township and country); origin of organic resources (animal, agro-industrial, urban, and mixture of urban and animal); nature (manure, agro-industrial waste, and compost); farm type (smallholder and agricultural factory); exploitation type (smallholder farm, factory farm, on-farm composting facility, and town composting facility); diversity of animals, animal feed, litter, sex, and age; diversity of bedding material; and diversity of OR evolution stage. The compositional analyses data include the sample identification number and the total organic matter, total organic carbon, inorganic matter, total nitrogen, phosphorus, calcium, magnesium, potassium, biochemical fractions (soluble, hemicellulose, cellulose, lignin and cutin), and contents of cadmium, chromium, copper, lead, nickel, and zinc.

The dataset is available via Cirad Dataverse.

## 2 Experimental Design, Materials and Methods

### 2.1 Sample Collection

One thousand animal, urban and agro-industrial ORs were collected from 6 regions in Madagascar. Animal ORs were collected without litter (feces and urine), including 84 bovine slurries, 289 porcine slurries, and 91 poultry droppings. Animal ORs were also collected as manure, including a mixture of animal feces and various plant materials used as feed or animal bedding. These ORs included 124 bovine manures, 13 ovine manures, 11 porcine manures, 245 poultry manures, 1 rabbit manure, and 1 mixture of bovine and porcine manures. A total of 71 urban ORs were collected from landfills. One digestate from a biodigester, 1 commercial lombricompost, and 8 potting soils completed the urban OR dataset. The agroindustrial OR consisted of 27 slaughterhouse residues (1 bovine bone, 13 bovine horn and 13 dried blood), 3 fishery residues, 3 tobacco ashes, 1 cotton shell from a cotton mill, 1 commercial fertilizer based on sugarcane residues, and 1 groundnut cake from an oil mill.

The collection procedure was adapted according to the nature and size of the sites (which ranged from small barns to large municipal solid waste landfills). The procedure consisted of the selection of representative zones, adaptation of the subsample number to be collected (a minimum of 3 to 20 subsamples) and the creation of composite samples from subsamples.

The samples were rapidly transported to the laboratory and stored at 4°C until preparation for NIR or compositional analyses.

### 2.2 Chemical Analysis Methods

A subsample of each sample was air-dried and ground to a size of 0.2 mm using a porcelain mortar and pestle set before analysis.

The total organic carbon (TOC) and total nitrogen (TN) were measured using a Flash EA1112 elementary analyzer (Thermo Finnigan, San Jose, USA), and the total organic matter (TOM) was determined by loss on ignition at 550°C according to NF V18.101 [3].

The total phosphorus (totP), potassium (totK), magnesium (totMg), and calcium (totCa) were determined after calcination of aliquotes (520°C) and ash solubilization in hydrochloric acid. The totK, totCa and totMg were quantified by using an iCE 3000 atomic absorption spectrometer (Thermo Fisher Scientific, Waltham, USA), and the totP was determined by spectrophotometry using a continuous flow analyzer with an 810 Uvikon spectrophotometer (Kontron, Eching, Germany).

For trace element analyses, a representative OR subsample was ground to a size of 100 μm before dissolution. After calcination at 450°C, total dissolution was performed by acid digestion using a mixture of HF, HNO_3_ and HClO_4_ [4]. The concentrations were then determined with a Vistra-PRO (Varian, Palo Alto, USA) inductively coupled plasma-optical emission spectrometer using an axially viewed plasma system and a charge-coupled device detector. For quality control, in-house reference samples and certified samples (CRM 7001 Light Sandy Soil and CRM 7004 Loam, Analytica) were used every 20 samples, and each analysis was conducted in duplicate.

The OR biochemical composition was determined using the Van Sœst method [1] as modified under the French standard FD U 44-162 [5]. The soluble (SOL), hemicellulose-like (HCEL) cellulose-like (CEL) and lignin and cutin-like (LIC) fractions were separated. After each extraction step, the OM content in the residues was determined by loss on ignition at 550°C, and the ash-free biochemical fractions were expressed as g 100 g^−1^ dry weight.

### 2.3 Spectroscopic Analysis

The VIS-NIR spectra were recorded using a Labspec spectrometer (ASD, Boulder, USA). The OR samples were packed in ring cups and scanned in reflectance mode. Two different spectra (each averaged from 32 scans) were recorded for each sample using independent cup fillings in terms of the absorbance (the logarithm of the inverse of reflectance). Both absorbance spectra were then averaged until good repeatability was obtained, based on the root mean square (RMS) value; i.e., the RMS between two sample spectra was less than 2.5 times the average of all the RMS values calculated for each sample of the dataset. If good repeatability was not achieved, spectrum acquisition was repeated.

The OR VIS-NIR spectra were recorded on a LabSpec spectrometer (ASD, Boulder, USA). Standard cell spectra were acquired using the LabSpec spectrometer and an XDS spectrometer (Foss, Silver Spring, USA). The spectra obtained using the LabSpec spectrometer were standardized using a method described by Gogé *et al.* [2], taking the XDS spectrometer as the master instrument. The mean spectra are reported in Fig. 4.

## Ethics Statement

Not applicable

## CRediT authorship contribution statement

**Nantenaina Rabetokotany Rarivoson:** Conceptualization, Methodology, Data curation, Writing – original draft, Writing – review & editing. **Tantely Razafimbelo:** Supervision, Validation, Writing – review & editing, Project administration. **Dominique Masse:** Supervision, Validation, Writing – review & editing, Project administration. **Heriniaina Ramahefarison:** Methodology, Data curation, Validation, Writing – review & editing. **Laurent Thuriès:** Conceptualization, Methodology, Data curation, Supervision, Writing – review & editing, Project administration.

## Declaration of Competing Interest

The authors declare that they have no known competing financial interests or personal relationships that have or could be perceived to have influenced the work reported in this article.

## Data Availability

Spectroscopic (VIS-NIR) analysis of organic resources with animal, agro-industrial, and urban origins collected from different regions of Madagascar (Original data) (Dataverse). Compositional analysis of organic resources with animal, agro-industrial, and urban origins collected from different regions of Madagascar (Original data) (Dataverse).

## Appendix Supplementary materials

Image, application 1

Image, application 2

Image, application 3

Image, application 4

Image, application 5

Image, application 6

Image, application 7
